# microRNA-203 inhibits migration and invasion of canine tonsillar squamous cell carcinoma cells by targeting *SLUG*

**DOI:** 10.3389/fvets.2023.1239224

**Published:** 2023-08-03

**Authors:** Shunsuke Noguchi, Asuka Matsui

**Affiliations:** ^1^Laboratory of Veterinary Radiation, Graduate School of Veterinary Science, Osaka Metropolitan University, Osaka, Japan; ^2^Laboratory of Veterinary Radiology, College of Life, Environment, and Advanced Sciences, Osaka Metropolitan University, Osaka, Japan

**Keywords:** dog, microRNA-203, migration and invasion, SLUG, squamous cell carcinoma

## Abstract

**Objective:**

Squamous cell carcinoma (SCC) occurring in the tonsils (TSCC) has a poorer prognosis than SCC occurring in other regions of the oral cavity (non-tonsillar SCC [NTSCC]) because it easily metastasizes to distant organs. This study aimed to elucidate the molecular mechanisms underlying the migration and invasion of TSCC cells *in vitro*.

**Materials and methods:**

This study focused on differential microRNA (miRNA) expression using microRNA microarrays and quantitative polymerase chain reaction in canine TSCC and NTSCC tissues and cell lines. A target gene of the miRNA involved in cell migration and invasion was validated by wound healing, transwell, and luciferase assays.

**Results:**

miR-203 expression was lower in TSCC tissues than in the normal oral mucosa and NTSCC tissues. Transfection of the miR-203 mimic resulted in the downregulation of mesenchymal marker protein expression and attenuation of cell migration and invasion in TSCC cells, but not in NTSCC cells. A dual-luciferase assay revealed that miR-203 directly targeted the mesenchymal transcription factor *SLUG*. SLUG overexpression enhances the migration of TSCC cells.

**Conclusion:**

Our study indicates that the miR-203/SLUG axis may be involved in the metastatic mechanisms of TSCC.

## 1. Introduction

Squamous cell carcinoma (SCC) is one of the most common oral cavity cancers. Canine oral SCC (CoSCC) primarily develops in the mucosa of the gingiva (non-tonsillar SCC; NTSCC) and tonsils (TSCC). NTSCC has a high potential for invasion of the surrounding stroma and adjacent osseous tissues, whereas the rate of metastasis to regional lymph nodes and distant organs is relatively low (at most 36%) (1). Local recurrence is a major concern in NTSCC treatment. In contrast, the prognosis of TSCC is worse than that of NTSCC because of the high metastasis rate (at most 73%) (1, 2). Thus, treating CoSCC is often challenging for veterinarians, and the molecular mechanisms underlying its invasion or metastasis remain unclear.

Epithelial mesenchymal transition (EMT) is important in cell migration and invasion (3). During EMT, nonmotile epithelial cells, such as carcinoma cells, change their phenotype to motile mesenchymal-like cells. The transitioned cells can invade the fibrous stroma and vessels around the primary lesion, resulting in metastasis to distant organs. EMT is regulated by mesenchymal transcription factors, such as zinc finger E-Box Binding Homeobox 1, TWIST, SNAIL, and SLUG (4). In our previous study, SLUG has been suggested to be upregulated in canine oral SCC and is associated with the migration and invasion of TSCC and NTSCC cells (5).

Many microRNAs (miRNAs and miRs) are associated with EMT in normal and cancer cells (6–8). However, only a few studies on the association between miRNAs and EMT have been published in veterinary medicine (9–11). Although EMT-associated miRNAs have not been validated in CoSCC, miR-145 suppresses the migration of CoSCC cells by targeting *Fascin 1* (12).

In this study, we focused on the differential expression of miRNAs and validated their functions to reveal their roles in the mechanisms underlying EMT induction, cell migration, and invasion in TSCC.

## 2. Materials and methods

### 2.1. Tissues and cell lines

Canine oral SCC cell lines (TSCCLN#1–#6; TSCC cell lines and oSCC-1, -3, and -4; NTSCC cell lines), which we had established, were cultured under the conditions described previously (13). Viable cells were counted using the trypan blue dye exclusion test. All tested cell lines were validated to be canine origin by partial genome sequencing and tested for mycoplasma contamination using the EZ-PCR Mycoplasma Test Kit (Biological Industries, Beit Haemek, Israel). No contaminants were detected.

Six normal oral mucosa tissues, 12 NTSCC tissues, eight TSCC tissues, six TSCC cell lines, and three NTSCC cell lines were used for total RNA extraction (Table 1). All tissues were surgically excised for treatment or biopsy, and experienced pathologists diagnosed SCC. This study was approved by the authors’ institutional ethics committee (approval number: R01-001).

**Table 1:** Clinical specimens used for total RNA extraction.

Sample number	Breed	Sex	Age (yr)	Location	WHO clinical stage
1	Chihuahua	SF	13	Tonsil	III
2	Miniature Dachshund	M	13	Tonsil	III
3	Miniature Dachshund	M	12	Tonsil	I
4	Toy poodle	CM	11	Tonsil	III
5	Border Collie	CM	7	Tonsil	III
6	Chihuahua	CM	14	Tonsil	II
7	CKCS	CM	11	Tonsil	II
8	Chihuahua	CM	11	Tonsil	IV
9	Toy poodle	M	12	Maxillary	III
10	Toy poodle	CM	7	Maxillary	III
11	Pekingese	FS	10	Maxillary	III
12	Spitz	FS	9	Maxillary	III
13	Labrador Retriever	FS	15	Maxillary	I
14	Toy poodle	FS	12	Maxillary	I
15	Shiba	CM	12	Mandibular	III
16	Labrador Retriever	FS	13	Mandibular	II
17	Labrador Retriever	CM	10	Mandibular	III
18	Toy poodle	CM	16	Mandibular	II
19	Pug	M	11	Mandibular	II
20	Toy poodle	FS	13	Buccal	III

M, Male; CM, Castrated male; F, Female; SF, Spayed female.

CKCS, Cavalier King Charles Spaniel.

WHO, World Health Organization.

### 2.2. microRNA microarray

Total RNA was extracted from a normal oral mucosa tissue, SCC tissues (sample number 1 and 9), oSCC-1, and TSCCLN#6 cells and was processed by microRNA microarray analysis using the GeneChip^™^ miRNA array. The data were analyzed using the Microarray Data Analysis tool (Filgen, Inc., Aichi, Japan), and miRNAs showing >2-fold differential expression are indicated in a heatmap.

### 2.3. Quantitative reverse transcription-polymerase chain reaction using real-time PCR

Total RNA was extracted from cells using the phenol/guanidinium thiocyanate method with DNase I treatment. To analyze the expression level of miRNAs, we performed TaqMan MicroRNA Assays (hsa-miR-203 and *RNU44*; Thermo Fisher Scientific, Waltham, MA, United States) following reverse transcription using the TaqMan MicroRNA Reverse Transcription Kit (Thermo Fisher Scientific). Real-time PCR using THUNDERBIRD^®^ Probe qPCR Mix (TOYOBO, Osaka, Japan) was performed. The relative expression level of miR-203 was analyzed by the ΔΔCt method. *RNU44* was used as an internal control.

### 2.4. Reagents and antibodies

The miR-203 mimic (mirVana^™^ miRNA mimic) and miR-203 inhibitor (mirVana^™^ miRNA inhibitor) were purchased from Thermo Fisher Scientific. According to the manufacturer’s protocol, cationic liposomes with Lipofectamine RNAiMAX (Invitrogen) were used for transfection. Pre-miR miRNA precursor molecule-negative control #2 (Applied Biosystems, Foster City, CA, United States) was used as a non-specific miRNA control.

The following rabbit monoclonal antibodies were used: anti-E-cadherin antibody (#3195, 1:1000, Cell Signaling Technology, Denvers, MA, United States), anti-Vimentin antibody (#5741, 1:1000, Cell Signaling Technology), anti-phosphorylated SMAD3 (p-SMAD3) antibody (#9520, 1:1000, Cell Signaling Technology), anti-SMAD3 antibody (#9513, Cell Signaling Technology), anti-Slug antibody (#9585, 1:1000, Cell Signaling Technology), and anti-Snail antibody (#3879, 1:1000, Cell Signaling Technology). Horseradish peroxidase-conjugated secondary antibodies were obtained from Cell Signaling Technology. Anti-β-actin mouse monoclonal antibody (Sigma-Aldrich, St. Louis, MO, United States) was used as a loading control.

A canine SLUG expression plasmid harboring the open reading frame of canine *SLUG* (accession number; XM_038441070.1) was constructed using the pIRESpuro3 plasmid vector (Takara). The pIRESpuro3 empty vector was used as a negative control (mock). Transfection was performed using Lipofectamine 3000 (Invitrogen) in accordance with the manufacturer’s protocol. The cells were seeded into 6-well plates at 1.0 × 10^5^/well concentration. After seeding for 24 h, the plasmids were transfected at a concentration of 4 μg/well.

### 2.5. Western blotting

As previously described, total protein was extracted from whole cells (14). Protein concentrations were measured by the Bradford method using Bio-Rad Protein Assay Reagent Concentrate (Bio-Rad, Hercules, CA, United States). Ten micrograms of protein lysates were separated using sodium dodecyl sulfate-polyacrylamide gel electrophoresis. The proteins were electroblotted onto polyvinylidene fluoride membranes (PerkinElmer Life Sciences, Boston, MA, United States). The details of the method used after blotting have been previously described (14). The antibodies were diluted in the appropriate buffer. Immunoblots including the loading control were visualized using a Lightning ECL Pro (PerkinElmer Life Sciences).

### 2.6. Wound healing assay

A total of 1.0 × 10^5^ cells/well were spread in 6-well plates. Transfection with miRNAs or plasmid vectors was performed the following day. When 90% confluence was achieved, wounds were made using the head of a 200 μL tip. The detailed procedure was described previously (12).

### 2.7. Cell invasion assay

The invasive ability of all tested cell lines was estimated using the CytoSelect 24-well Cell Invasion Assay Kit (Cell BioLabs, Inc., San Diego, CA, United States) according to the manufacturer’s protocol. 1.0 × 10^5^ cells/well were spread in 6-well plates. miRNAs were transfected on the next day. After 24 h of transfection, 3.0 × 10^5^ cells were displaced in the transwell system and incubated for 48 h. The number of invading cells was measured under a microscope after staining with the solution in the kit.

### 2.8. Dual luciferase assay

Using TargetScan,^1^ *SLUG* and *SMAD3* were identified as miR-203 targets. The 3′-UTR of canine *SLUG* or *SMAD3*, which included the biding site of miR-203, was amplified using LA Taq (Takara, Shiga, Japan) with the following primers: *SLUG*, sense; 5′-CTG ACA GCT AGA TTG AGA GG, antisense; 5′-GCC AAT AAG GAG TAT GCA CC, and *SMAD3*, sense; 5′-AAT TGC AGG CTT GGT GCA GA, antisense; 5′-GCA GGT CTG TAT CTC AAA TG. A sensor vector was constructed by combining the amplified region with the luciferase reporter pMIR-control vector (Ambion, Foster City, CA, United States). In addition, to generate the sensor vectors with mutations in the binding site for miR-203, seed regions were mutated from CATTTCA to GCAATGC using a PrimeSTAR^®^ Mutagenesis Basal Kit (Takara, Otsu, Japan). The sequence of the mutated sensor vector was confirmed by Fasmac (Atsugi, Japan). The cells were seeded in 12-well plates at a concentration of 0.5 × 10^5^/well the day before transfection. The sensor vector at a concentration of 1.0 μg/well and 40 nM miR-203 or non-specific control miRNA were co-transfected into the cells using Lipofectamine RNAiMAX. At 48 h after co-transfection, the luciferase activity was measured using the Dual-Glo^™^ Luciferase Assay System (Promega, Madison, WI, United States) following the manufacturer’s instruction. The luciferase activity of firefly was normalized to that of *Renilla*.

### 2.9. Statistics

Each experiment was performed in triplicate. All data were compared using an unpaired 2-tailed Student’s *t*-test or one-way analysis of variance (ANOVA) following Tukey’s method after evaluation for data distribution. All statistical analyses were performed using the Microsoft Excel add-in software Statcel 4 (OMS Publication, Tokyo, Japan). A *p*-value of < 0.05 was considered statistically significant.

## 3. Results

### 3.1. miR-203 was downregulated in SCC tissues and cell lines

**Figure 1:**
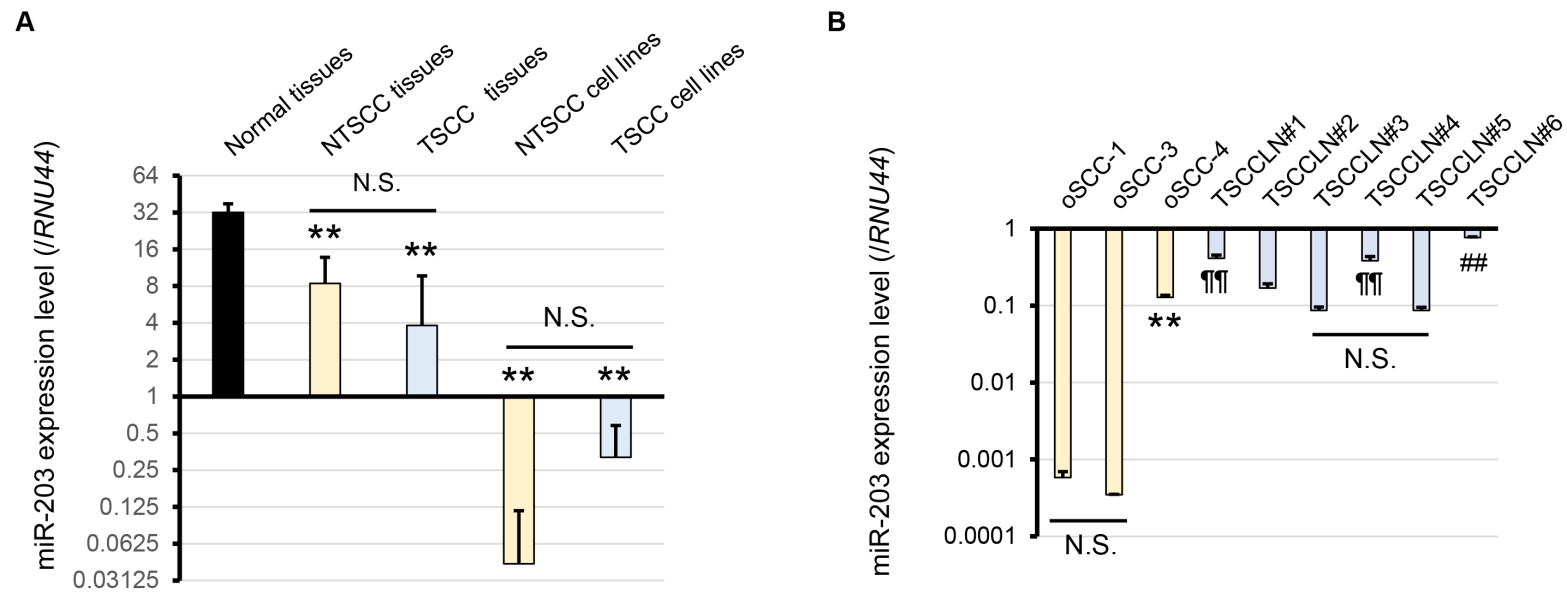
**(A)** Expression level of miR-203 analyzed by real-time PCR using a TaqMan probe in normal tissues, NTSCC tissues, TSCC tissues, NTSCC cell lines (oSCC-1, -3, and -4), and TSCC cell lines (TSCCLN#1-#6). **p < 0.01, for difference vs. normal tissues assessed by one-way ANOVA following Tukey’s test. N.S. indicates no significance. **(B)** Expression level of miR-203 in each cell line. **, ¶¶, and ##*p* < 0.01, for differences among oSCC cell lines (**), vs. TSCCLN#3 and #5 (¶¶), and vs. other TSCCLN cell lines (##). N.S. indicates no significance. Data are expressed as the mean + SD (*n* = 3).

We focused on miRs-32, 203, 205, 574, 708, and 1841 based on the results of miRNA microarray analysis (Supplementary Figure S1). Of these, miR-203 alone was consistently downregulated in NTSCC, TSCC, NTSCC cell lines, and TSCC cell lines, and miR-205 was also downregulated in these cell lines (Figure 1A; Supplementary Figure S2). Furthermore, the expression of miR-203 in oSCC-4 and TSCCLN#6 cells was much higher than that in the other NTSCC and TSCC cell lines (Figure 1B). miR-203 expression in TSCCLN#1 and #4 cells was intermediate, whereas that in TSCCLN#3 and #5 cells was the lowest among the TSCC cell lines. Based on these results, we evaluated the function of miR-203 in the NTSCC and TSCC cell lines.

### 3.2. miR-203 decreased the expression of the mesenchymal markers

**Figure 2:**
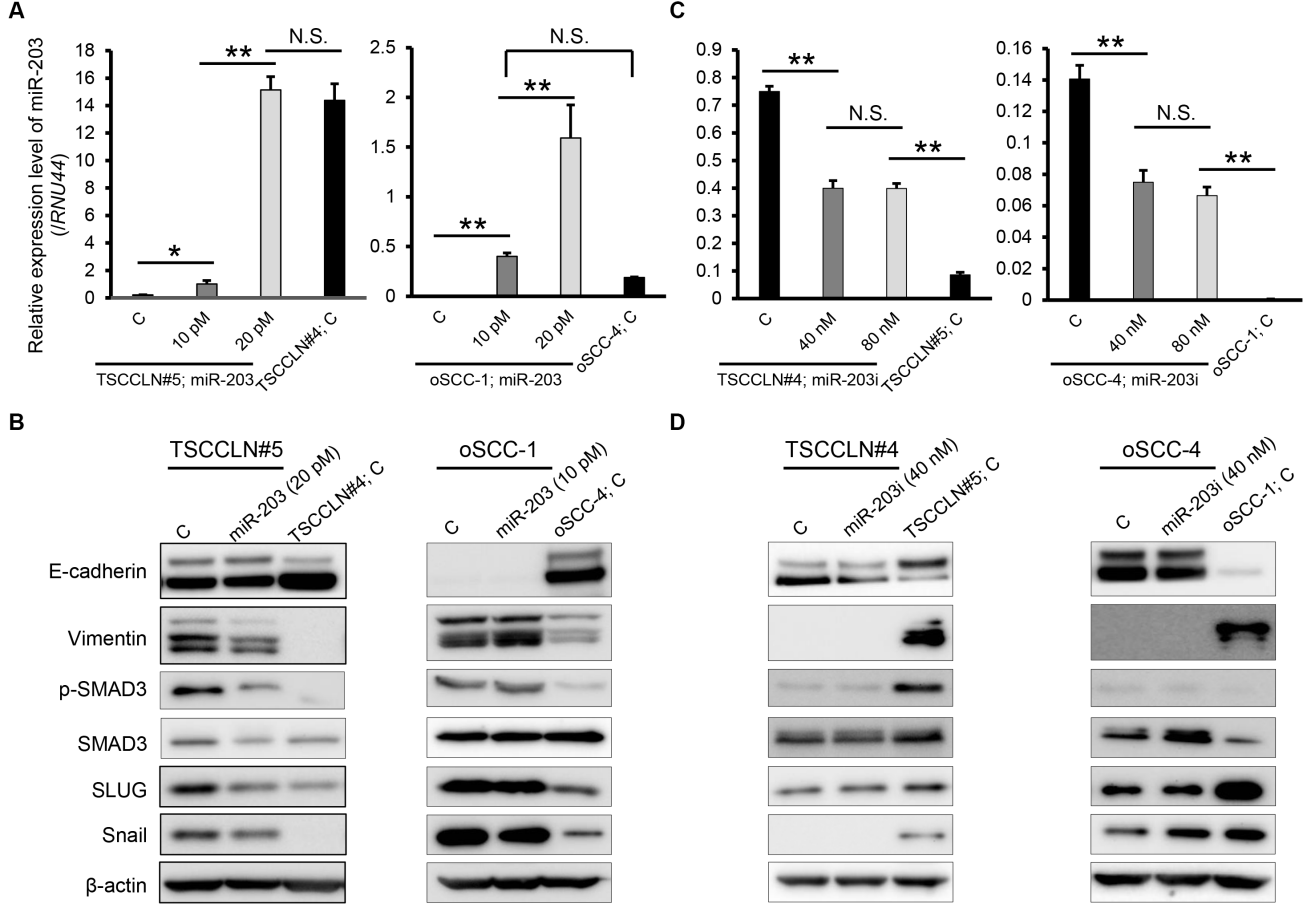
Expression level of miR-203 **(A)** and protein expression levels of EMT markers including the putative targets of miR-203 **(B)** in TSCCLN#5 and oSCC-1 cells transfected with a miR-203 mimic at indicated doses and TSCCLN#4 and oSCC-4 cells transfected with a negative control miRNA mimic. Expression level of miR-203 **(C)** and protein expression levels of EMT markers including the putative targets of miR-203 **(D)** in TSCCLN#4 and oSCC-4 cells transfected with a miR-203 inhibitor at indicated doses and TSCCLN#5 and oSCC-1 cells transfected with a negative control miRNA inhibitor. **p < 0.01, for differences between the samples connected with a line or bracketed. N.S. indicates no significance. Data are expressed as the mean + SD (*n* = 3).

The miR-203 mimic was transfected into TSCCLN#5 and oSCC-1 cells with the relatively lower miR-203 expression levels among all the cell lines (Figure 1B), at 10 or 20 pM doses. The expression levels of miR-203 in cells transfected with 20 pM (TSCCLN#5) and 10 pM (oSCC-1) were similar to those in TSCCLN#4 and oSCC-4 cells, which showed the most prominent epithelial phenotypes among the TSCCLN and oSCC cell lines (Figure 2A) (13). Transfection with miR-203 decreased the expression of mesenchymal markers (vimentin, p-SMAD3, SMAD3, SLUG, and Snail) in TSCCLN#5 cells, but not in oSCC-1 cells (Figure 2B; Supplementary Figure S3A). In contrast, inhibition of miR-203 decreased the expression of E-cadherin in TSCCLN#4 cells, but not in oSCC-4 cells. However, the miR-203 inhibitor did not sufficiently suppress miR-203 expression (Figures 2C,D; Supplementary Figure S3B). These results indicated that miR-203 is associated with EMT in TSCC cells. In contrast, transfection with miR-203 mimic at 20 pM did not affect the viability of TSCCLN#5 or oSCC-1 cells (Supplementary Figure S4).

### 3.3. miR-203 inhibited the migration and invasion of TSCCLN cells

**Figure 3:**
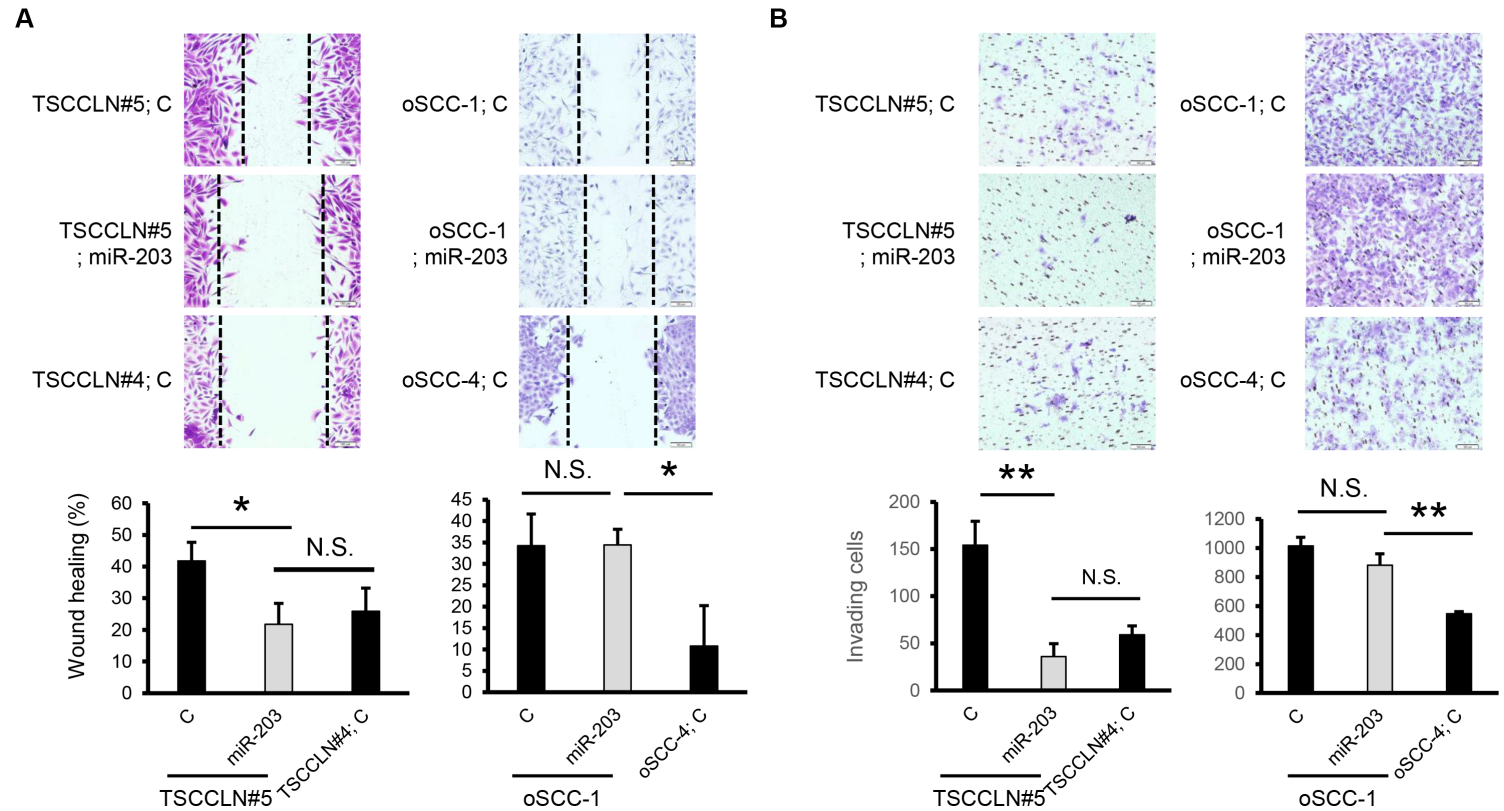
Effects of miR-203 on cell migration and invasion. **(A)** Cell migration assessed by wound healing assay in the cells transfected with miR-203 mimic or the negative control miRNA at a dose of 20 pM (TSCCLN#5) or 10 pM (oSCC-1). **(B)** Cell invasion assessed by Transwell assay in the cells transfected with miR-203 mimic or the negative control miRNA at a dose of 20 pM (TSCCLN#5) or 10 pM (oSCC-1). *p < 0.05 and **p < 0.01, for differences between the samples connected with a line. N.S. indicates no significance. Data are expressed as the mean + SD (*n* = 3).

We performed a wound healing assay to evaluate cell migration. Transfection with miR-203 mimic significantly suppressed the migration of TSCCLN#5 cells to the level observed in TSCCLN#4 cells (Figure 3A). However, migration suppression was not observed in oSCC-1 cells transfected with the miR-203 mimic. In the transwell invasion assay, similar to the results of the wound healing assay, miR-203 significantly inhibited the invasion of TSCCLN#5 cells to the level of TSCCLN#4 cells but not that of oSCC-1 cells (Figure 3B).

### 3.4. Slug was a target of miR-203 and played a pivotal role in the migration of TSCC cells

**Figure 4:**
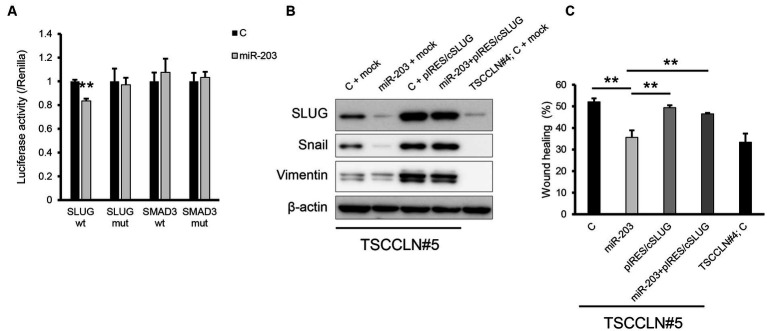
Validation of the target gene of miR-203. **(A)** Dual luciferase assay for *SLUG* and *SMAD3*. Wt indicates the sensor vector harboring the wild-type sequences of the target site of miR-203 and mut indicates that harboring the mutated sequences of the target site of miR-203. **p < 0.01, difference between the luciferase activity of the cells transfected with control miRNA and that of those with miR-203 mimic. Data are expressed as the mean + SD (*n* = 3). **(B)** Protein expression of the mesenchymal markers in TSCCLN#4 and #5 cells transfected with the indicated miRNA and/or plasmid vector. **(C)** Cell migration assessed by wound healing assay in TSCCLN#4 and #5 cells transfected with the indicated miRNA and/or the plasmid vector. **p < 0.01, difference between the samples connected with a line. Data are expressed as the mean + SD (*n* = 3).

A dual-luciferase assay revealed that miR-203 significantly decreased the luciferase activity of the pMIR reporter vector harboring wild-type sequences of the 3′-UTR of *SLUG* but not the mutated 3′-UTR (Figure 4A). In contrast, miR-203 did not decrease the luciferase activities of the plasmid vector harboring wild-type or mutated sequences of 3′-UTR of *SMAD3*. Overexpression of SLUG using a plasmid harboring the *SLUG* ORF upregulated the expression of mesenchymal markers and consistently promoted the migration of TSCCLN#5 cells, regardless of the combined transfection with miR-203 (Figures 4B,C; Supplementary Figure S5). These results indicated that miR-203 may regulate the migration and invasion of TSCC cells by targeting *SLUG*.

## 4. Discussion

Validation of the mechanisms involved in TSCC metastasis is required to improve the prognosis. This study revealed that miR-203 is downregulated in both TSCC and NTSCC and targets *SLUG*, which plays an important role in sustaining the mesenchymal phenotype, resulting in the suppression of the migration and invasion of TSCC cells but not those of oSCC cells (5).

miR-203 was downregulated in CoSCC tissues including TSCC, compared to normal tissues, and its expression level in SCC cell lines was markedly downregulated compared to that in SCC tissues, indicating that stromal components in tumor tissues had a relatively high expression of miR-203. The results of this and previous studies suggest that miR-203 may be a commonly downregulated miRNA in canine cancers (15–17).

We used TSCCLN#4 and #5 cell lines as TSCC cells in this study. TSCCLN#4 cells showed relatively high miR-203 expression and an epithelial phenotype, whereas TSCCLN#5 cells showed low miR-203 expression and a mesenchymal phenotype (13) (Figure 1C). miR-203 affects the mesenchymal epithelial transition in TSCCLN#5 cells via the downregulation of SLUG but not in TSCCLN#4 cells. Therefore, we hypothesized that miR-203 functions in cells with a mesenchymal phenotype. However, the effect of miR-203 was not observed in oSCC cells, and the reason for this remains unclear. These results indicate that the miR-203/SLUG axis is involved in the metastatic mechanisms of TSCC.

Our results indicate that *SMAD3* is not a direct target of miR-203, despite the decreased protein expression of SMAD3 in TSCCLN#5 cells transfected with the miR-203 mimic, although *SMAD3* has been suggested to be a target of miR-203 in human lung cancer (18). miR-203 might repress TGF-β/SMAD3 signaling indirectly in canine TSCC cells, resulting in a decrease in SMAD3 expression, as miR-203 and TGF-β/SLUG signaling can form a double negative feedback loop to suppress one another’s expression (19).

In the current study, we failed to validate the effects of miR-203 on metastasis *in vivo*. We attempted to develop a metastatic model by transplanting several TSCCLN and oSCC cell lines into the tongue and gingiva of immunodeficient mice, respectively. However, metastasis to distant organs was not observed. In the future, a metastatic rodent model should be developed to clarify the effects of miR-203.

## 5. Conclusion

The current study suggests that miR-203 suppresses cell migration and invasion through targeting *SLUG* in TSCC cells, in which miR-203 expression is markedly downregulated but not in NTSCC cells.

## Data availability statement

The original contributions presented in the study are included in the article/Supplementary material, further inquiries can be directed to the corresponding author.

## Ethics statement

The animal studies were approved by the ethics committee of Veterinary Medical Center of Osaka Metropolitan University. The studies were conducted in accordance with the local legislation and institutional requirements. Written informed consent was obtained from the owners for the participation of their animals in this study.

## Author contributions

SN and AM conceived and planned the experiments and carried out the experiments. AM contributed to sample preparation. SN contributed to the interpretation of the results and took the lead in writing the manuscript. All authors contributed to the article and approved the submitted version.

## Funding

This work was supported by JSPS KAKENHI (Grant Number 20K06435).

## Conflict of interest

The authors declare that the research was conducted in the absence of any commercial or financial relationships that could be construed as a potential conflict of interest.

## Publisher’s note

All claims expressed in this article are solely those of the authors and do not necessarily represent those of their affiliated organizations, or those of the publisher, the editors and the reviewers. Any product that may be evaluated in this article, or claim that may be made by its manufacturer, is not guaranteed or endorsed by the publisher.

## Footnotes

^1^ http://www.targetscan.org/vert_72/
