# Supplementary material for: microRNA-203 inhibits migration and invasion of canine tonsillar squamous cell carcinoma cells by targeting SLUG
Source: Front Vet Sci. 2023 Aug 3;10:1239224. doi: 10.3389/fvets.2023.1239224 (PMC10434855; doi:10.3389/fvets.2023.1239224)
Supplement: Supplementary file 1 [file Image_1.PDF]

Supplementary figure 1

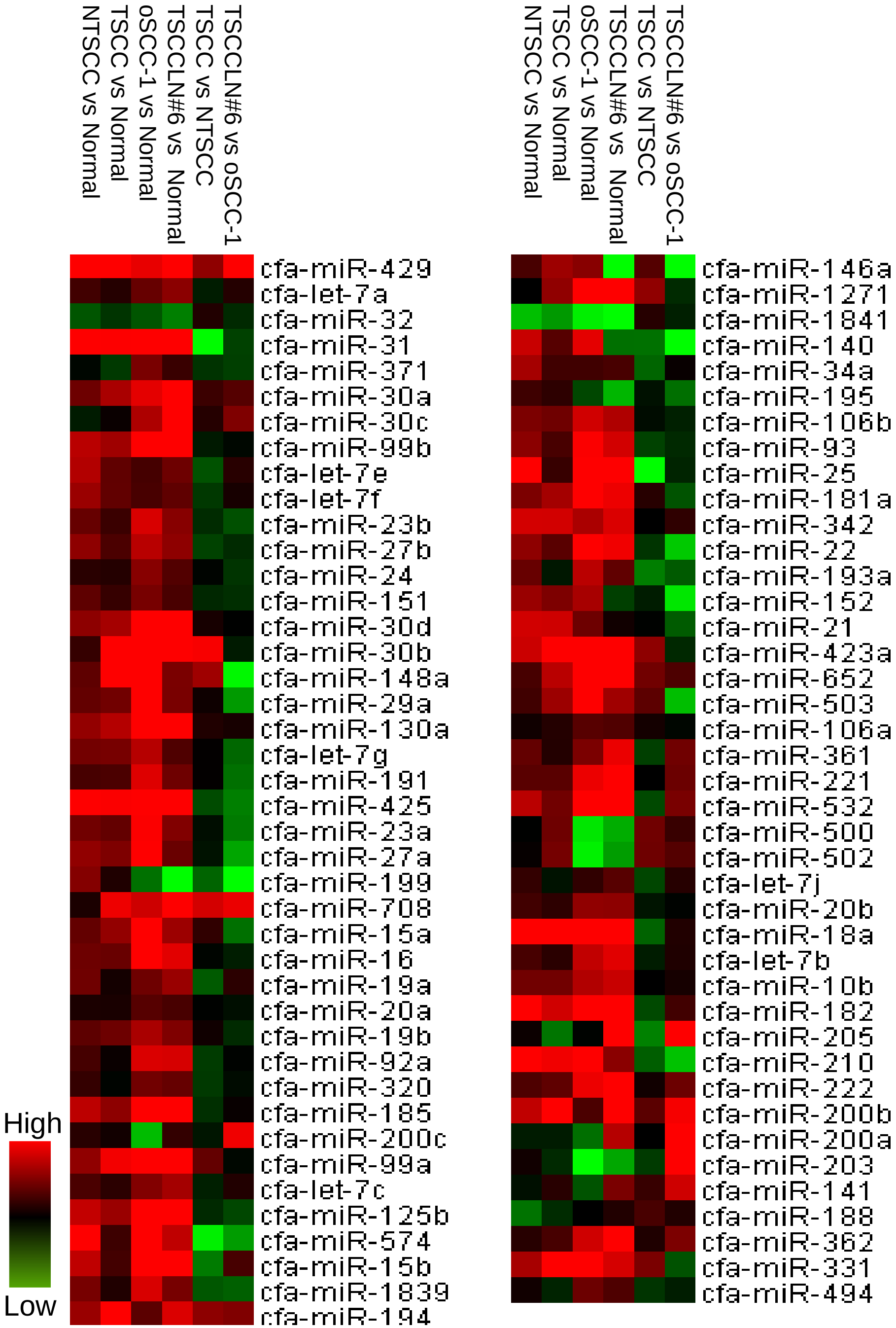

A heatmap of microRNA microarray using an NTSCC tissue (sample number 9), a TSCC tissue (sample number 4), TSCCLN#6 cells, and oSCC-1 cells.

Total RNA was extracted from a normal oral mucosa tissue, SCC tissues (sample number 1 and 9), oSCC-1, and TSCCLN#6 cells and was processed by microRNA microarray analysis using the GeneChip^{TM} miRNA array. The data were analyzed using the Microarray Data Analysis tool (Filgen, Inc., Aichi, Japan), and miRNAs showing >2-fold differential expression and obvious expression are indicated in a heatmap.

Supplementary figure 2

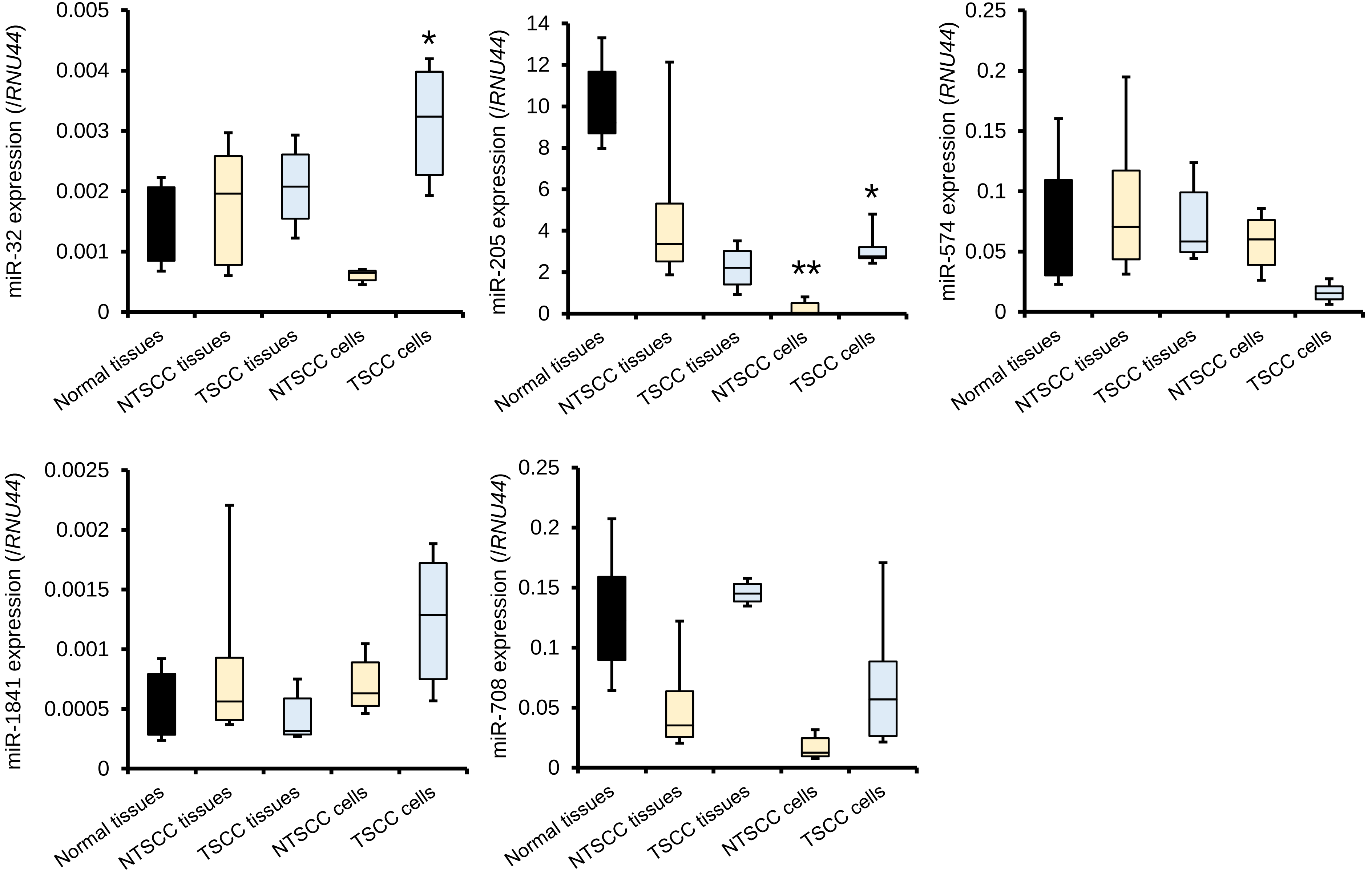

Comparative miRNA expressions among indicated tissues and cell lines. Statistic analysis was performed using one-way ANOVA following Tukey method. *$p < 0.05$, **$p < 0.01$.

Supplementary figure 3

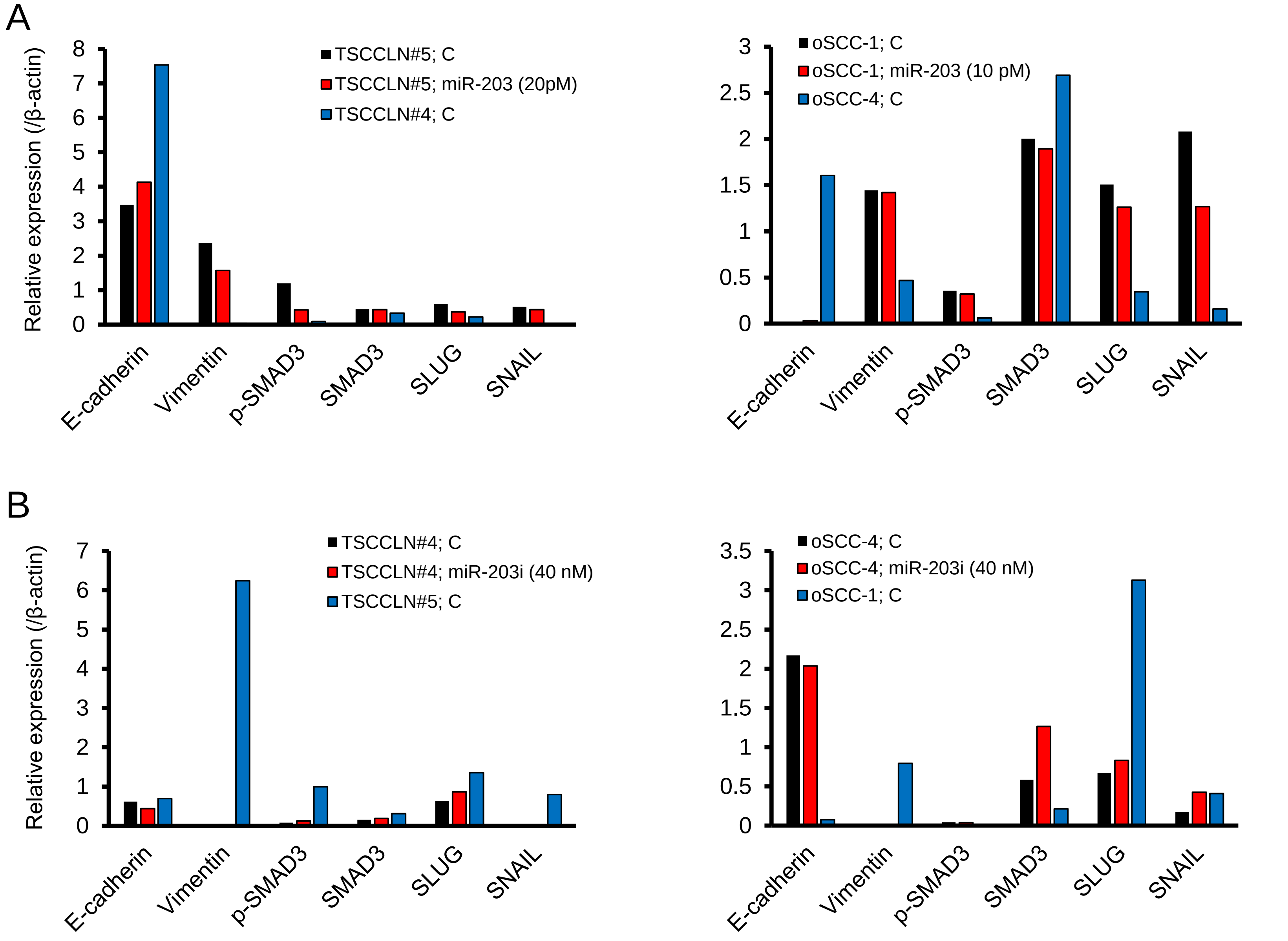

Densitometric values of protein expression indicated in Fig. 2B (**A**) and D (**B**). Each value was normalized to the value of β-actin.

Supplementary figure 4

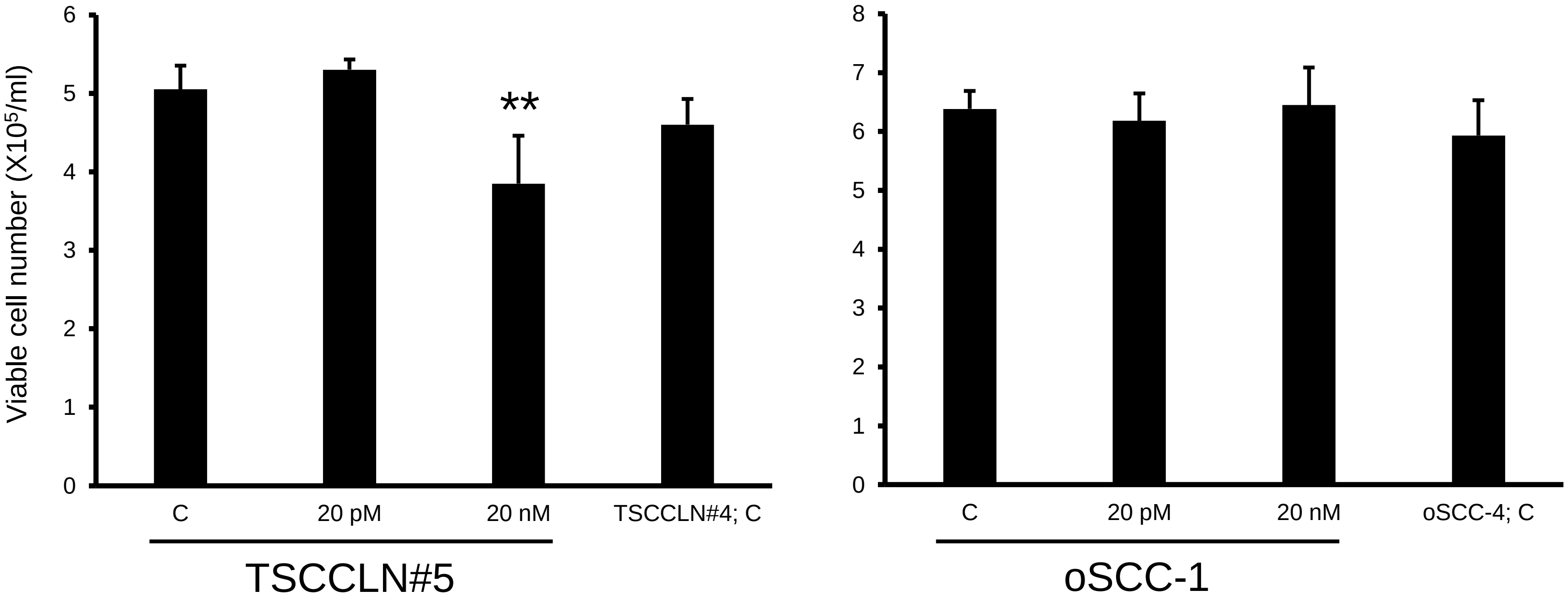

The number of viable cells 48 h after the transfection with miR-203 mimic at an indicated dose. Statistic analysis was performed using one-way ANOVA following Tukey method. ***p* < 0.01.

Supplementary figure 5

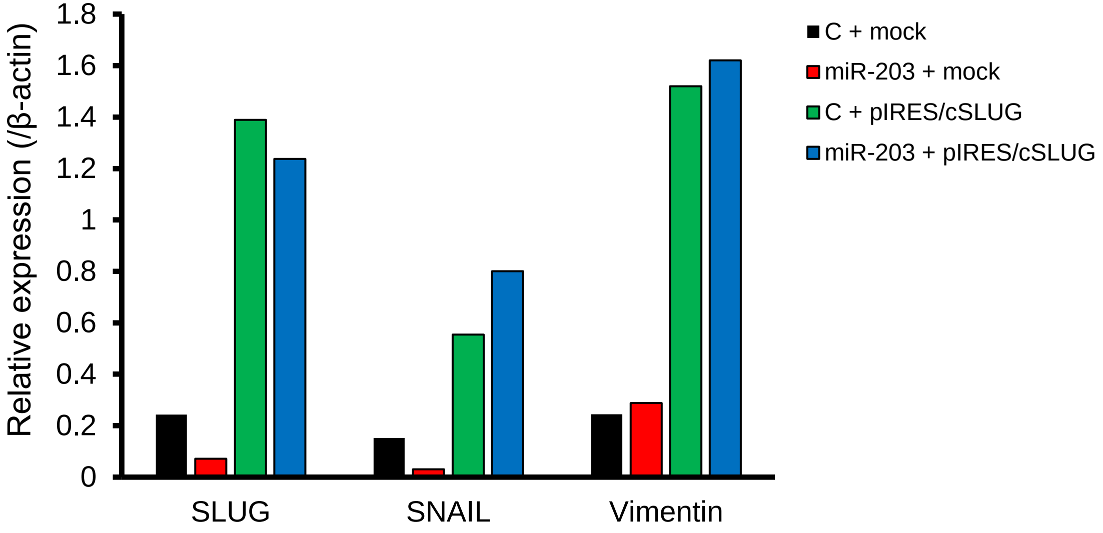

Densitometric values of protein expression indicated in Fig. 4B. Each value was normalized to the value of β-actin.
